# Marker Placement Reliability and Objectivity for Biomechanical Cohort Study: Healthy Aging in Industrial Environment (HAIE—Program 4)

**DOI:** 10.3390/s21051830

**Published:** 2021-03-05

**Authors:** Jan Malus, Jiri Skypala, Julia Freedman Silvernail, Jaroslav Uchytil, Joseph Hamill, Tomas Barot, Daniel Jandacka

**Affiliations:** 1 Human Motion Diagnostic Center, Department of Human Movement Studies, University of Ostrava, 70200 Ostrava, Czech Republic; jiri.skypala@osu.cz (J.S.); jfs@unlv.edu (J.F.S.); jaroslav.uchytil@osu.cz (J.U.); jhamill@kin.umass.edu (J.H.); daniel.jandacka@osu.cz (D.J.); 2 Department of Kinesiology and Nutrition Sciences, University of Nevada Las Vegas, Las Vegas, NV 89154, USA; 3 Department of Kinesiology, University of Massachusetts, Amherst, MA 01003, USA; 4 Department of Mathematics with Didactics, University of Ostrava, 70900 Ostrava, Czech Republic; tomas.barot@osu.cz

**Keywords:** standard error of measurement, minimal detectable change, multi-segment foot model, optoelectronic stereophotogrammetry, lower limb

## Abstract

In large cohort studies, due to the time-consuming nature of the measurement of movement biomechanics, more than one evaluator needs to be involved. This may increase the potential occurrence of error due to inaccurate positioning of markers to the anatomical locations. The purpose of this study was to determine the reliability and objectivity of lower limb segments length by multiple evaluators in a large cohort study concerning healthy aging in an industrial environment. A total of eight evaluators performed marker placements on five participants on three different days. Evaluators placed markers bilaterally on specific anatomical locations of the pelvis, thigh, shank and foot. On the right foot, markers were placed in anatomical locations to define a multi-segmental foot model. The position of the marker at the anatomical locations was recorded by a motion capture system. The reliability and objectivity of lower limb segment lengths was determined by the intraclass correlation coefficient of a two-way random model and of the two-way mixed model, respectively. For all evaluators for all segments, the average reliability and objectivity was greater than 0.8, except for the metatarsus segment (0.683). Based on these results, we can conclude that multiple evaluators can be engaged in a large cohort study in the placement of anatomical markers.

## 1. Introduction

Musculoskeletal health status is one of the main goals of preventive strategies from a healthy aging perspective. These strategies focus on an active lifestyle to reduce the impact of obesity, cardiovascular diseases, malignancy, bone health and diabetes. Musculoskeletal diseases, however, can have adverse effects as they limit the ability of individuals to make changes towards an active lifestyle [1]. The success of the medical system has increased longevity with the result that the general population is living longer with accompanying chronic musculoskeletal diseases [2]. To understand these musculoskeletal health conditions, biomechanics research studies should focus on an epidemiologically reasonable size of the population. In the past decade, prospective studies have utilized approximately 10 to 500 participants when analyzing running [3,4,5] and 10 to 150 when analyzing walking [6,7,8]. Recently, there has been an acceleration in the progress of measurement technologies and data analysis, making it possible to obtain much larger sample sizes for epidemiological research purposes. Correct placement of the anatomical markers is necessary for further biomechanical analysis. However, the main problem persists with the objectivity of marker placement for kinematic movement analysis.

In large cohort studies, it is necessary to involve more than one evaluator due to the time-consuming nature of the motion capture measurement [9]. The project from which these data are taken (HAIE—Program 4, www.4haie.cz, accessed on 20 January 2021) is a large-scale research study and will investigate the potential impact of physical activity in highly polluted air on musculoskeletal health [10]. Unfortunately, the large participant pool has the potential to increase the number of errors caused by inaccurate marker placement when more than one evaluator is involved [11]. It has been shown that the kinematic data of gait is affected by up to 75% due to human error [12]. On the other hand, it has been also shown that the second evaluator does not substantially impact the interclass correlation coefficient or the minimal detectable change values for kinematic and kinetic measures of gait [13]. A key element in motion caption analysis is to identify the length of the segment at the beginning of the measurement [14]. The length of the segment is a parameter in many calculations of the kinematics and kinetics of gait [15]. However, previous methodological studies have focused only on the resulting gait kinematics [9,16,17].

Therefore, investigation into whether the placement of markers by different evaluators can affect reliability and objectivity is necessary. This is especially important in quantitative projects with a large sample size such as the HAIE project (1500 participants). For these large-scale projects, it is common to use more than one evaluator which can lead to an increased chance of human error. Therefore, it is necessary to determine the objectivity to make the measured data relative. The present technique is well known in motion capture systems [9,12,16,18,19,20,21]. Earlier research examined relatively small samples of the population, so there was no need to involve a larger number of examiners in the measurements. With technological advances, such as the automatic identification of markers, did it become financially affordable to carry out measurements on large cohorts. Thus, we need to standardize the potentially largest source of error. We have to point out that this marker placement technique is used in other motion capture system [22,23,24] and also in MRI measurement [25]. The novelty and purpose of this study was to determine the reliability and objectivity of the lower limb segment length on different days by multiple evaluators using a motion capture system. We hypothesized that a value greater than 0.8 for reliability and objectivity for each segment would be found with data from the HAIE project. We also anticipated that the standard error measurement would be less than 5%. The results of this research can be useful in all systems that need to identify significant points of the human body with a multiple number of examiners on different days by retroreflective markers.

## 2. Methods

### 2.1. Participants

Three males and two females, who were not part of the HAIE project, were used as participants in this study. They served as models for marker placement. The HAIE project divided the population into active and inactive participants according to a certain criteria [10]. In total, three of them were classified as active and two were classified as inactive. The basic anthropometric data (age, height, weight and body mass index) of the participants were 28.6 ± 9.3 y, 1.77 ± 0.2 m; 79.8 ± 20.6 kg; and 25.6 ± 4.8 kg/m^2^, respectively.

### 2.2. Experimental Set-Up

Eight evaluators participated in marker placement within the biomechanical measurement of project HAIE. The evaluators (M1 to M8) had 4 to 6 years’ experience (approximately 50–300 participants) with palpating participants within their research studies. Each evaluator graduated in the human movement analysis field and obtained a license in physiotherapy. Evaluators M2 and M6 were also trained in marker placement and analysis of human movement capture at the Department of Kinesiology, University of Massachusetts, Amherst, MA, USA.

A motion capture system was used to detect retroreflective markers by ten infrared cameras (9× Oqus 700+, 1× Oqus 510+, Qualisys, Inc., Gothenburg, Sweden) located around the lab at a height of 2.5 m. A sampling frequency of 240 Hz was used to record the marker position data. Before each measurement, a global coordinate system was calibrated with a wand calibration kit based on Qualisys recommendation (Qualisys, Inc., Gothenburg, Sweden).

### 2.3. Protocol

At the beginning of the measurement, participants wore neutral laboratory running shoes (Brooks Launch 5, Brooks Sport Inc., Seattle, WA, USA) predetermined for use in the HAIE project. To determine the reliability and objectivity of marker placement and consequently segment length, each evaluator placed retroreflective markers on five participants on three different days. In total, 32 individual retroreflective markers and four marker cluster plates containing four fixed markers (9.5 mm diameter Pearl Markers) were attached bilaterally to specific anatomical locations of the pelvis, thigh, shank and foot. Each evaluator placed retroreflective calibration and tracking markers on the pelvis bilaterally and on the anterior and posterior superior iliac spines. Markers were placed on the right and left greater trochanters of the femur, the medial and lateral femoral condyles, and the medial and lateral malleoli. Marker cluster plates were also placed on the thigh and shank [26]. On the left foot, markers were placed on the head of the first and fifth metatarsal heads along with three markers placed on the heel. On the right foot, the markers were placed on the most distal and dorsal point of the head of the proximal phalanx of the hallux, the head and base of the first, second and fifth metatarsals, the most medial apex of the tuberosity of the navicular, the most medial apex of the sustenaculum tali, the lateral apex of the peroneal tubercle and triad markers on the heel [27,28]. The participant then stood at a specifically labelled position in the calibration space during which a standing calibration trial was recorded.

## 3. Data Analysis

Markers in the standing calibration trial were labeled according to the recommendation in Qualisys Track Manager software (Qualisys, Sweden). Visual 3D v6 (C-Motion, Rockville, MD, USA) was used to create a skeletal model of the pelvis and the lower extremity. The pelvis segment was modeled as a cylinder and the foot, thigh, and shank segments as right circular cones [29]. The hip joint center was specified according to the C-Motion recommendation with a radius correction, which was defined as the distance between the hip joint center and the greater trochanter of the femur [30]. The knee joint center position was defined as the mid-point between the medial and lateral femoral epicondyles, while the ankle joint center was defined as the mid-point of the medial and lateral malleoli [31].

**Figure 1:**
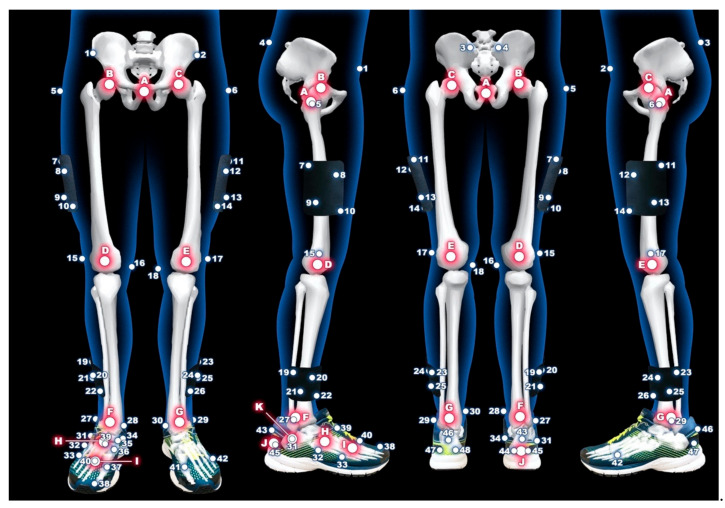
Marker placement model of lower extremities.

**Figure 2:**
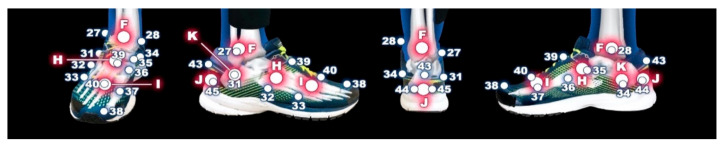
Marker placement multi-segmental model.

Pelvis length was created by calibration and target the markers right and left anterior superior iliac spine, right and left posterior superior iliac spine. Thigh length segment was defined as the distance between the hip joint center and the knee joint center. Shank length segment was defined as the distance between the knee joint center and the ankle joint center. The length of the foot was determined as the distance between the ankle joint center and the mid-point between the head of the first and fifth metatarsals. Two landmarks were created to define the length of the calcaneus. The distal part of the calcaneus was defined as the center between two lower points on the calcaneus (Figure 1 and Figure 2, Table 1). The end of the proximal part was defined as the mid-point between the most medial apex of the sustenaculum tali and the lateral apex of the peroneal tubercle. Mid-foot length was defined as the distance between base of the second metatarsal and the joint center determined by most medial apex of the tuberosity of the navicular and base of the fifth metatarsal. Metatarsus length was determined as the distance between base of the second metatarsal and the joint center created from heads of the first and fifth metatarsals [27,32].

## 4. Statistical Analysis

Lower limb segment length reliability and objectivity were determined by the intraclass correlation coefficient of the two-way random model (ICC_2,1_) and the interclass correlation coefficient of the two-way mixed model (ICC_3,k_), respectively [33,34]. In addition, for reliability and objectivity, the standard error measurement (SEM), the minimal detectable change (MDC), and their percentage of the mean were calculated [33]. For lower limb segment length reliability, the mean, standard deviation (SD), SEM, %SEM, MDC, and %MDC were calculated for each evaluator for each segment from the five participants from three standing calibration trials. The mean, SD, SEM, %SEM, MDC, and %MDC were averaged from 8 evaluators for each segment. For lower limb segment length objectivity, the ICC was calculated from all three standing calibration trials and from each standing calibration trial. The presented mean, SD, SEM, %SEM, MDC, and %MDC were calculated from the average value of individual final values from five participants from 8 evaluators. ICC were interpreted based on the following classifications: less than 0.5 poor, between 0.5 and 0.75 moderate, between 0.75 and 0.9 good, and more than 0.90 excellent [35]. All data were calculated using the IBM SPSS Statistics 24 (IBM SPSS Inc., Chicago, IL, USA).

For the considered data, which are necessary for the computation of the further provided arithmetical averages of evaluators, the testing the normality was consistently obtained always as *p* ≥ *α*. The achieved *p*-values can be seen in Table 2. The significance value *α* was declared as 0.001. For the purposes of the proof of the normality of data, the Shapiro-Wilk test was chosen and applied in the frame of the statistical software SPSS. For data, which fulfills the property of the normal probability distribution, the following determination of the reliability criterion can be suitable expressed by using the *z*-scores, which can be utilized for the normal distributed data.

## 5. Results

The average reliability of the lower limb segment length of all evaluators for all segments was greater than 0.8, except for the right metatarsus segment (0.683). Table 3 shows the reliability of the lower limb segment length of individual evaluators. Most of the segments except multi-segmental foot had a reliability greater than 0.9, except for evaluators M1 (left thigh = 0.620), M4 (right thigh = 0.856) and M7 (left thigh = 0.890 and right thigh = 0.815). The lowest ICC were from evaluators M1 and M5 on the right metatarsus (0.402 and 0.318) and from evaluator M4 on the right calcaneus (0.322). The largest %SEM and %MDC of marker placement was 6.26% and 17.35% on the right metatarsus of the multi-segment foot (Table 4).

Lower limb segment length objectivity was greater than 0.8 for all evaluators for all segments. Table 5 presents the objectivity of lower limb segment length in individual rounds. The lowest objectivity score of the lower limb segment length was in the right metatarsus segment, both in single standing calibration trials and for all records (0.694–0.860). The %SEM was less than 4% and the %MDC was less than 9% (Table 6).

## 6. Discussion

The aim of this study was to assess the importance of marker placement reliability and objectivity using motion analysis data. For a project that deals with a large cohort, where measurements are undertaken by more than one evaluator, it is necessary to determine the reliability and objectivity of the data. It was hypothesized that evaluators with an average of five years of experience would be able to achieve a higher value than 0.8 for reliability and objectivity for each segment. This study confirmed, on average, higher reliability and objectivity values greater than 0.8 for every segment except for the right metatarsus.

The first part of our study was to determine reliability. Although the marker placement was performed on three different days, we achieved high reliability with all evaluators. This finding does not necessarily mean that the results of the resulting kinematic and kinetic data will also be reliable. Nevertheless, based on previous research focused on the reliability of gait analysis, we assume that the position marker data estimated by our evaluators is highly reliable [10,12]. However, we must take into account that the resulting values were influenced by the application of markers, the identification of anatomical landmarks, data processing, the laboratory setting, and especially by the natural variability of human factors [34,35]. In addition, it needs to be emphasized that the segment length is entered as the main parameter for calculating the kinematic and kinetic data of a gait analysis. Hence, we decided to choose only the length of the segment. Therefore, it is of utmost importance to know that similar results can be achieved by repeatedly determining the length of the lower limb segments. According to the result of determining the segment length of individual evaluators, the reliability was between 0.75 and 0.9, signifying ‘good’ reliability [35]. The question remains whether individual evaluators are able to determine the length of the segments similarly.

The results of the second part of objectivity were surprisingly consistent even though eight different evaluators were used in our study which is more than what has been evaluated in previous studies [11]. In the current study, a “strong” objectivity was achieved for every segment except the right metatarsus. Based on previous research that focused on the objectivity of gait analysis [9,11], we can assume that the resulting kinematic and kinetic data would also be highly reliable. Despite the high experience of evaluators with marker placement, meta-tarsal segment had a lower reliability and objectivity, which is probably due to less experience with marker placement at the multi-segment foot. The standard deviation of the calibration is the difference between the actual wand length and the length perceived by the cameras. Therefore, it is best that the standard deviation in the calibration is a very small number. Values of the standard deviation of wand length in our calibrations are in the range of 0.5–0.8 mm. These values are very low and will have a negligible effect on the segment length results than can occur with evaluator error. The obtained data were measured in the Qualisys Track manager (Qualisys, Sweden), which is one of several leading analysis systems in the field of motion capture. According to a comparative study, it is possible to generalize the resulting values for other motion capture systems [36,37,38,39,40].

The main limitation of this study is that we focused on only one parameter (segment length). In this study, we did not analyze the orientation of the segment in the measurement which is another limitation. The limitations of the software and hardware play an integral part of the present data, but it is a negligible factor when comparing of errors caused by the human placement of markers and soft tissue movement. Typical errors of motion capture systems are less than 1 mm [12,38]. Consequently, in repeated measurements of gait analysis capture, we are not able to distinguish the errors between measurement and natural biological variability [41]. On the other hand, the strength of this research is the relatively high number of evaluators and data collected from three different days. Further studies, which take other parameters into account, should be undertaken.

## 7. Conclusions

Eight different evaluators determined similar segment lengths by repeatedly measuring the size of the segments. We achieved “good” reliability and objectivity of segment length determination at all except one lower extremity segment and moderate reliability and objectivity at the metatarsus segment. From the current study, therefore, it can be concluded that multiple evaluators may be utilized in large cohort motion analysis studies. This work may contribute to the pre data collected phase of large biomechanical studies.

## Figures and Tables

**Table 1:** The legend for Figure 1 and Figure 2.

**Number**	**Anatomical Location**	**Number**	**Anatomical Location**
1	right anterior superior iliac spine	29	left lateral malleoli of ankle
2	left anterior superior iliac spine	30	left medial malleoli of ankle
3	left posterior superior iliac spine	31	right lateral apex of the peroneal tubercle
4	right posterior superior iliac spine	32	right base of the fifth metatarsal
5	right great trochanter of the femur	33	right head of the fifth metatarsal
6	left great trochanter of the femur	34	right most medial apex of the sustentaculum tali
7–10	right marker cluster plate on the thigh	35	right most medial apex of the tuberosity of the navicular
11–14	left marker cluster plate on the thigh	36	right base of the first metatarsal
15	right lateral femoral condyle of knee	37	right head of the first metatarsal
16	right medial femoral condyle of knee	38	head of the proximal phalanx of the hallux
17	left lateral femoral condyle of knee	39	right base of the second metatarsal
18	left medial femoral condyle of knee	40	right head of the second metatarsal
19–22	right marker cluster plate on the shank	41	left head of the first metatarsal
23–26	left marker cluster plate on the shank	42	left head of the fifth metatarsal
27	right lateral malleoli of ankle	43–45	triad markers on the right heel
28	right medial malleoli of ankle	46–48	triad markers on the left heel
**Letter**	**Landmark**	**Letter**	**Landmark**
A	center between no.5 and no.6	G	left ankle joint center
B	right hip joint center	H	center between no.32 and no.35
C	left hip joint center	I	center between no.33 and no.37
D	right knee joint center	J	center between no.44 and no.45
E	left knee joint center	K	center between no.31 and no.34
F	right ankle joint center		

**Table 2:** Achieved Results of Testing Normality of Data Using Shapiro-Wilk Test.

	*p*-Values of the Shapiro-Wilk Test									
	PEL	RTH	LTH	RSK	LSK	RF	LF	RC	RMF	RM
M1	0.277	0.820	0.122	0.009	0.302	0.446	0.335	0.107	0.582	0.274
M2	0.095	0.485	0.074	0.013	0.034	0.691	0.305	0.619	0.039	0.054
M3	0.027	0.079	0.101	0.031	0.025	0.527	0.606	0.873	0.675	0.459
M4	0.093	0.259	0.126	0.085	0.028	0.286	0.681	0.813	0.044	0.525
M5	0.025	0.109	0.320	0.032	0.009	0.347	0.512	0.356	0.607	0.271
M6	0.080	0.380	0.308	0.001	0.004	0.928	0.359	0.108	0.891	0.832
M7	0.010	0.288	0.098	0.011	0.132	0.244	0.446	0.654	0.666	0.568
M8	0.067	0.222	0.125	0.005	0.051	0.613	0.202	0.097	0.128	0.420

PEL—Pelvis; RTH—Right thigh; LTH—Left Thigh; RSK—Right shank; LSK—Left shank; RF—Right Foot; LF—Left foot; RC—Right calcaneus; RM—Right metatarsus; RMF—Right mid-foot

**Table 3:** Reliability of lower limb segments length. Mean intraclass correlation computed from all records of each segment by eight evaluators (M1–M8). ICC_mean was calculated from eight evaluators.

Segment	M1_ICC	M2_ICC	M3_ICC	M4_ICC	M5_ICC	M6_ICC	M7_ICC	M8_ICC	ICC_Mean
PEL	0.972	0.968	0.994	0.982	0.986	0.985	0.988	0.956	0.979
RTH	0.931	0.959	0.983	0.856	0.991	0.972	0.815	0.983	0.936
LTH	0.620	0.954	0.975	0.958	0.974	0.967	0.890	0.978	0.915
RSK	0.982	0.995	0.985	0.971	0.986	0.989	0.989	0.992	0.986
LSK	0.979	0.977	0.986	0.977	0.986	0.975	0.992	0.984	0.982
RF	0.957	0.982	0.988	0.986	0.995	0.980	0.995	0.982	0.983
LF	0.982	0.980	0.981	0.985	0.993	0.986	0.984	0.987	0.985
RC	0.851	0.882	0.897	0.322	0.914	0.855	0.936	0.771	0.804
RM	0.402	0.818	0.541	0.848	0.318	0.924	0.839	0.770	0.683
RMF	0.913	0.884	0.931	0.697	0.924	0.947	0.862	0.960	0.890

ICC—Intraclass correlation coefficient; PEL—Pelvis; RTH—Right thigh; LTH—Left Thigh; RSK—Right shank; LSK—Left shank; RF—Right Foot; LF—Left foot; RC—Right calcaneus; RM—Right metatarsus; RMF—Right mid-foot.

**Table 4:** Reliability of lower limb segments length. Mean (X), standard deviation (SD), standard error measurement (SEM), minimal detectable change (MDC), and their percentage of the mean from eight evaluators.

Segment	X (cm)	SD (cm)	SEM (cm)	MDC (cm)	%SEM	%MDC
PEL	13.52	1.39	0.19	0.54	1.43	3.95
RTH	43.21	2.53	0.54	1.50	1.25	3.46
LTH	43.11	2.55	0.65	1.79	1.49	4.12
RSK	40.93	2.76	0.31	0.87	0.77	2.13
LSK	40.74	2.77	0.36	1.01	0.90	2.48
RF	13.80	1.26	0.16	0.43	1.13	3.12
LF	13.94	1.26	0.15	0.43	1.10	3.05
RC	6.08	0.91	0.36	1.01	6.08	16.85
RM	5.78	0.71	0.36	1.00	6.26	17.35
RMF	5.91	0.59	0.18	0.51	3.14	8.69

PEL—Pelvis; RTH—Right thigh; LTH—Left Thigh; RSK—Right shank; LSK—Left shank; RF—Right Foot; LF—Left foot; RC—Right calcaneus; RM—Right metatarsus; RMF—Right mid-foot.

**Table 5:** Objectivity of lower limb segments length. Mean interclass correlation coefficient computed from three single standing calibration trials and from all records of each segment by eight evaluators.

Segment	ICC1	ICC2	ICC3	ICC	95% Confidence Interval	
					Lower Bound	Upper Bound
PEL	0.990	0.990	0.990	0.995	0.984	0.999
RTH	0.943	0.965	0.988	0.981	0.938	0.998
LTH	0.979	0.968	0.989	0.990	0.968	0.999
RSK	0.994	0.991	0.991	0.996	0.987	1.000
LSK	0.993	0.991	0.988	0.995	0.985	0.999
RF	0.990	0.997	0.995	0.998	0.993	1.000
LF	0.993	0.992	0.994	0.997	0.989	1.000
RC	0.948	0.951	0.935	0.975	0.917	0.997
RM	0.805	0.798	0.694	0.860	0.541	0.984
RMF	0.942	0.937	0.943	0.971	0.903	0.997

ICC—Interclass correlation coefficient; PEL—Pelvis; RTH—Right thigh; LTH—Left Thigh; RSK—Right shank; LSK—Left shank; RF—Right Foot; LF—Left foot; RC—Right calcaneus; RM—Right metatarsus; RMF—Right mid-foot.

**Table 6:** Objectivity of lower limb segments length. Mean (X), standard deviation (SD), standard error measurement (SEM), minimal detectable change (MDC), and their percentage of the mean from eight evaluators.

Segment	X (cm)	SD (cm)	SEM (cm)	MDC (cm)	%SEM	%MDC
PEL	13.52	0.39	0.03	0.08	0.20	0.57
RTH	43.21	1.19	0.16	0.45	0.38	1.05
LTH	43.11	0.92	0.09	0.26	0.21	0.59
RSK	40.93	1.07	0.07	0.19	0.16	0.46
LSK	40.74	0.87	0.06	0.17	0.15	0.42
RF	13.80	0.23	0.01	0.03	0.08	0.21
LF	13.94	0.22	0.01	0.03	0.09	0.24
RC	6.08	0.64	0.10	0.28	1.67	4.62
RM	5.78	0.47	0.18	0.49	3.03	8.40
RMF	5.91	0.53	0.09	0.25	1.54	4.27

PEL—Pelvis; RTH—Right thigh; LTH—Left Thigh; RSK—Right shank; LSK—Left shank; RF—Right Foot; LF—Left foot; RC—Right calcaneus; RM—Right metatarsus; RMF—Right mid-foot.
